# Combinations of modifiable lifestyle behaviours in relation to colorectal cancer risk in Alberta’s Tomorrow Project

**DOI:** 10.1038/s41598-020-76294-w

**Published:** 2020-11-25

**Authors:** Dylan E. O’Sullivan, Amy Metcalfe, Troy W. R. Hillier, Will D. King, Sangmin Lee, Joy Pader, Darren R. Brenner

**Affiliations:** 1 Department of Public Health Sciences, Queen’s University, Kingston, ON Canada; 2 Department of Community Health Sciences, University of Calgary, Calgary, AB Canada; 3 Department of Obstetrics & Gynecology, University of Calgary, Calgary, AB Canada; 4 Department of Medicine, University of Calgary, Calgary, AB Canada; 5 Department of Cancer Epidemiology and Prevention Research, CancerControl Alberta, Alberta Health Services, Holy Cross Centre – Room 513C, Box ACB, 2210-2nd St. SW, Calgary, AB T2S 3C3 Canada; 6 Department of Oncology, University of Calgary, Calgary, AB Canada

**Keywords:** Cancer epidemiology, Risk factors

## Abstract

The objective of this study was to identify distinct clusters of individuals that exhibit unique patterns of modifiable lifestyle-related behaviours and to determine how these patterns are associated with the risk of developing colorectal cancer (CRC). The study consisted of 26,460 participants and 267 CRC cases from Alberta’s Tomorrow Project. Exploratory latent class analysis of risk behaviours (obesity, physical inactivity, meat consumption, smoking, alcohol consumption, and fruit and vegetable consumption) and Cox proportional hazard models were utilized. Seven unique behavioural groups were identified, where the risk of CRC was 2.34 to 2.87 times greater for high risk groups compared to the low risk group. Sex-specific models identified higher risk groups among men (Hazard Ratios [HRs]: 3.15 to 3.89) than among women (HRs: 1.99 to 2.19). Targeting groups defined by clustering of behaviours could potentially lead to more effective prevention of CRC on a population level.

## Introduction

It is estimated that nearly one in two Canadians will be diagnosed with cancer in their lifetime and one in four will die of the disease^1^. Colorectal cancer (CRC) is the third most common cancer in Canada and is responsible for a large portion of the cancer-related mortality burden^1^. Non-modifiable risk factors for CRC include increasing age, male sex, a family history of colorectal cancer in a first degree relative, inflammatory bowel disease, and a personal history of colorectal adenomas^2,3^. CRC is also associated with several modifiable risk factors, and a recent study estimated that 43% of CRC cases in Canada were attributable to modifiable exposures – all of which were lifestyle behaviours^4^. The lifestyle-related exposures that have been consistently associated with CRC include obesity^5^, inadequate physical activity^5^, high consumption of red and processed meat^6^, low consumption of fruit and vegetables^5^, alcohol consumption^7^ and tobacco smoking^8^. Despite there being sufficient or probable evidence that several modifiable lifestyle behaviours are causally related to CRC, a large number of cases continue to be attributed to these factors, and the prevalence of many of these behaviours are not decreasing. For instance, in Canada smoking and physical inactivity are projected to decrease, while obesity, alcohol consumption, and poor dietary behaviours are projected to increase^4^.

It has been documented that engaging in certain lifestyle behaviours can increase the probability of engaging in other behaviours. For instance, smokers are also more likely to consume alcohol on a regular basis^9^. On the balance, there are also healthy behaviours that tend to occur with unhealthy behaviours. Individuals that engage in high levels of physical activity are more likely to consume alcohol^10^ and individuals that smoke are more likely to have a lower body mass index (BMI) – partly through decreased appetite stimulated by smoking^11^. Finally, there are other instances where paradoxical co-occurrences in behaviours can occur, such as individuals that engage in high levels of physical activity, but that also smoke^12^. The fact that individuals seldomly engage in a single behaviour can often make clear prevention messages challenging.

Given that resources for cancer prevention are limited, interventions targeting subgroups that share the same behaviours could be of value since they may be more cost-effective and could have a greater public health impact. To inform on the need for these types of programs, research on common patterns of behaviours is required. In addition, multifactorial prevention programs should be targeting exposure patterns that are common and that are associated with a high risk of disease, which necessitates examining the relationship of patterns of exposure with the risk of disease, such as CRC.

The examination of risk behaviour combinations necessitates a method of analysis that identifies the most common patterns of behaviours. Latent class analysis (LCA) is a statistical approach that utilizes subject values on a number of variables to identify homogeneous subgroups or classes of individuals that exist within a heterogeneous population^13^. Instead of identifying every possible profile, the LCA reduces the data in the most parsimonious set of classes. Each class has a unique behavioural profile and individuals are placed in a single class based on posterior probabilities^13^.

Several previous studies have used LCA to characterize clustering of modifiable behavioral risk factors for disease. The majority of these studies have been focused on specific populations, such as veterans^14^, college students^15^, adolescents^16^, or children^17^. Two studies were focused on general populations of the United States^18^ and the United Kingdom^19^, while one study was conducted on a sample of primary care patients^20^. To our knowledge, no study has used this method to characterize behaviours in Canada, and no study has examined clustering of colorectal cancer-specific risk behaviours. Importantly, no previous study has examined how classes determined by LCA are associated with the development of disease, which is an important consideration in the context of multifactorial intervention programs and resource management. The aim of this study was to determine distinct groups of individuals that exhibit unique patterns of lifestyle-related cancer risk factors in Alberta’s Tomorrow Project (ATP) cohort study and to determine how these groups were associated with the risk of CRC. In addition, we sought to explore the impact of sex and family history of CRC on patterns of these behaviours and subsequent risk of CRC. The overall goal of this investigation was to establish an LCA model for lifestyle behaviours and to illustrate its usefulness for establishing disease risk, enhancing population surveillance and in identifying possible interventions using CRC as an example.

## Results

### Study sample

The study sample with complete information on all relevant CRC risk factors and important demographic variables included 26,460 participants (9,892 males and 16,568 females). Only 78 (0.3%) of the 26,538 participants had missing data on one or more of the included variables and were excluded in downstream analyses. During a median follow-up of 13.23 years, 267 CRC cases (119 males and 148 females) occurred among the study participants. The characteristics of the study participants stratified by CRC status is summarized in Table 1. Characteristics of study participants stratified by sex and CRC status are presented in Supplemental Tables 1 and 2. Significant predictors of developing CRC in this study sample was having an overweight or obese BMI, being a current smoker, having a household income under $50,000, and having a family history of CRC. Among men, significant predictors of CRC included a high BMI, high risk alcohol consumption, being a current smoker, and having a family history of CRC. Among women, only being a current smoker was a significant predictor of developing CRC.

**Table 1:** Characteristics of participants in the Alberta Tomorrow Project and the risk of developing colorectal cancer status.

	Cases (n = 267)	Non-Cases (n = 26,193)	Hazard Ratio
	n (%)	n (%)	(95% CI)
**Follow-up time (years)**			
Mean (SD)	7.78 (4.2)	12.8 (3.64)	
**Age at baseline (years)**			
Mean (SD)	53.9 (8.6)	50.9 (9.2)	
**Sex**			
Female	148 (55.4)	16,420 (62.7)	1.0 (Ref)
Male	119 (44.6)	9773 (37.3)	1.23 (0.94–1.62)
**BMI**			
Normal	54 (20.2)	8935 (34.1)	1.0 (Ref)
Overweight	114 (42.7)	10,264 (39.2)	1.49 (1.07–2.07)
Obese	99 (37.1)	6994 (26.7)	1.82 (1.29–2.56)
**Fruit and vegetable consumption**			
Met guideline for both fruit and vegetable	139 (52.1)	12,282 (46.9)	1.0 (Ref)
Met guideline for fruit or vegetable	89 (33.3)	9974 (38.1)	0.85 (0.58–1.24)
Did not meet guideline for fruit or vegetable	39 (14.6)	3937 (15)	1.15 (0.80–1.66)
**Alcohol Consumption**			
Abstainer	45 (18.9)	4113 (15.7)	1.0 (Ref)
Low risk (under guidelines)	181 (67.8)	18,780 (71.7)	1.04 (0.75–1.45)
High risk (greater than guidelines)	41 (15.4)	3300 (12.6)	1.36 (0.88–2.11)
**Processed Meat**			
Less than 1 serving per week	140 (52.4)	14,916 (57)	1.0 (Ref)
1–2 servings per week	61 (22.9)	5619 (21.4)	1.19 (0.86–1.63)
Greater than 2 servings per week	66 (24.7)	5668 (21.6)	1.23 (0.88–1.70)
**Red Meat**			
Less than 3 servings per week	92 (34.5)	9055 (34.6)	1.0 (Ref)
3–6 servings per week	92 (34.5)	9817 (37.5)	0.92 (0.68–1.24)
Greater than 6 servings per week	83 (31.1)	7321 (28)	1.04 (0.73–1.48)
**Recreational Physical Activity**			
Greater than 300 min per week	91 (34.1)	11,010 (42)	1.0 (Ref)
150–300 min per week	73 (27.3)	6106 (23.3)	1.19 (0.8–1.61)
Less than 150 min per week	103 (38.6)	9077 (44.9)	0.91 (0.68–1.22)
**Tobacco Smoking**			
Never	86 (32.2)	11,747 (44.9)	1.0 (Ref)
Former	121 (45.3)	9885 (37.7)	1.28 (0.97–1.70)
Current	60 (22.5)	4561 (17.4)	1.63 (1.16–2.29)
**Ethnicity**			
Caucasian	251 (94)	23,777 (90.8)	1.0 (Ref)
Other	16 (6)	2416 (9.2)	1.27 (0.76–2.10)
**Household Income**			
$0-$49,999	123 (46.1)	8059 (30.8)	1.0 (Ref)
$50,000-$99,999	95 (35.6)	10,710 (40.9)	0.76 (0.57–1.00)
≥$100,000	49 (18.4)	7424 (28.3)	0.68 (0.48–0.97)
**Highest level of education**			
High school or less	96 (36)	7323 (28)	1.0 (Ref)
Some post-high school	53 (19.9)	5384 (20.5)	0.91 (0.65–1.27)
Post-secondary Degree	118 (44.2)	13,486 (51.5)	1.0 (0.76–1.32)
**Family History of CRC**			
No	227 (85)	24,055 (91.8)	1.0 (Ref)
Yes	40 (15)	2138 (8.2)	1.65 (1.17–2.31)

*Hazard ratios are mutually adjusted.

Abbreviations: BMI = body mass index; CRC = colorectal cancer; SD = standard deviation.

### Identification of latent classes

For the overall study sample, the Akaike information criterion (AIC), Bayesian information criterion (BIC), and sample size adjusted BIC decreased considerably until the 7-class model (Table 2). The AIC continued to decrease until the 10-class model, while the BIC and sample size adjusted BIC stopped decreasing at the 9-class model. The 8 and 9-class models only had marginally better fit than the 7-class model and did not elucidate a noticeably different “low risk class”. In addition, the 8 and 9-class models resulted in several classes with less than 10% of the study sample in the class. We therefore selected the 7-class model for this investigation. For sex-specific latent class analyses we selected the 6-class model for males and the 7-class model for females based on the same reasoning used for the overall model selection (Table 2).

**Table 2:** Model fit statistics for estimating classes of modifiable lifestyle-related cancer risk behaviours through exploratory latent class analysis.

Number of classes	AIC	BIC	Sample size adjusted BIC
Overall			
2	5925.54	6163.05	6070.88
3	4791.58	5151.93	5012.09
4	4399.51	4882.70	4695.20
5	3867.74	4473.78	4238.61
6	3505.84	4234.73	3951.89
7*	3242.97	4094.70	3764.19
8	3072.93	4047.50	3669.32
9	2929.67	4027.10	3601.25
10	2855.79	4076.06	3602.54
Men			
2	3321.20	3530.20	3438.05
3	3083.58	3400.69	3260.86
4	2818.77	3243.98	3056.49
5	2696.31	3229.63	2994.47
6*	2592.80	3234.23	2951.40
7	2494.84	3244.38	2913.88
Women			
2	4921.56	5145.47	5053.31
3	3939.49	4279.21	4139.38
4	3553.50	4009.03	3821.53
5	3299.50	3870.84	3635.67
6	3151.13	3838.28	3555.45
7*	2976.61	3779.58	3449.08
8	2889.35	3808.13	3429.95

### Cancer-risk behavioural profiles and the risk of colorectal cancer

**Figure 1:**
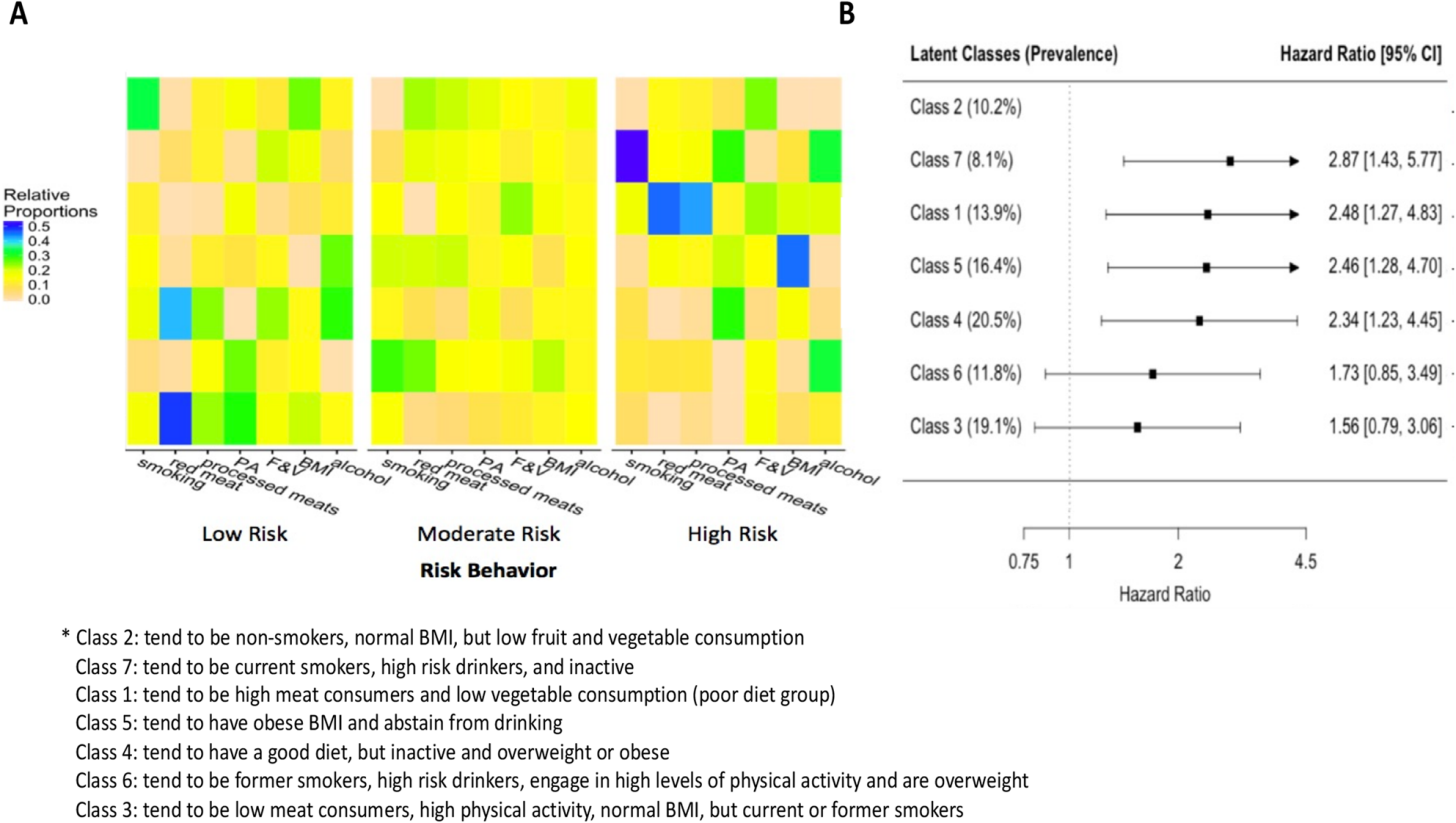
Latent class analysis of lifestyle-related colorectal cancer risk behaviours and the association of these classes with the risk of developing colorectal cancer. Panel A depicts the relative proportion of participants that belong in each category for each risk behaviour in each class. For each risk behaviour, the left is the low risk category and the right is the high risk category. Rows represent the latent classes and each column represents a category for a risk behaviour. Abbreviations: BMI = Body Mass Index, F & V = fruits and vegetables, PA = physical activity. Panel B depicts the risk (hazard ratios) of colorectal cancer for each class compared to the lowest risk class (class 2) adjusted for age, sex, ethnicity, household income, education, and family history of CRC.

The behavioural profiles of each latent class and the risk of developing CRC compared to the low risk class are displayed in Fig. 1A and 1B, respectively. The absolute probabilities for each risk behaviour for each latent class are presented in Supplemental Table 3. The low risk class (class 2) tended to consist of never smokers with normal BMI and low fruit and vegetable consumption. Compared to the low risk class, the classes with the highest risk of CRC were classes that tended to consist of individuals that engaged in low levels of physical activity, were current smokers, and engaged in high risk alcohol consumption (class 7; multivariable-adjusted hazard ratio [HR]: 2.87, 95% CI, 1.43–5.77); individuals that tended to have high meat consumption and low fruit and vegetable consumption (class 1; HR: 2.48, 95% CI, 1.27–4.83); individuals that tended to be obese and abstain from drinking alcohol (class 5; HR: 2.46, 95% CI, 1.28–4.70); and individuals that tended to engage in low levels of physical activity, have high fruit and vegetable consumption and low meat consumption (class 4; HR: 2.34, 95% CI, 1.23–4.45). The class that tended to consist of former smokers that were high-risk drinkers, engaged in high levels of physical activity and were overweight had a non-significant HR of 1.73 (class 6; 95% CI, 0.85, 3.49) compared to the low risk class. Compared to the low risk class, the class that tended to consist of individuals with low meat consumption, high physical activity, normal BMI and be a current or former smoker had an HR of 1.56 (class 3; 95% CI, 0.79, 3.06).

**Figure 2:**
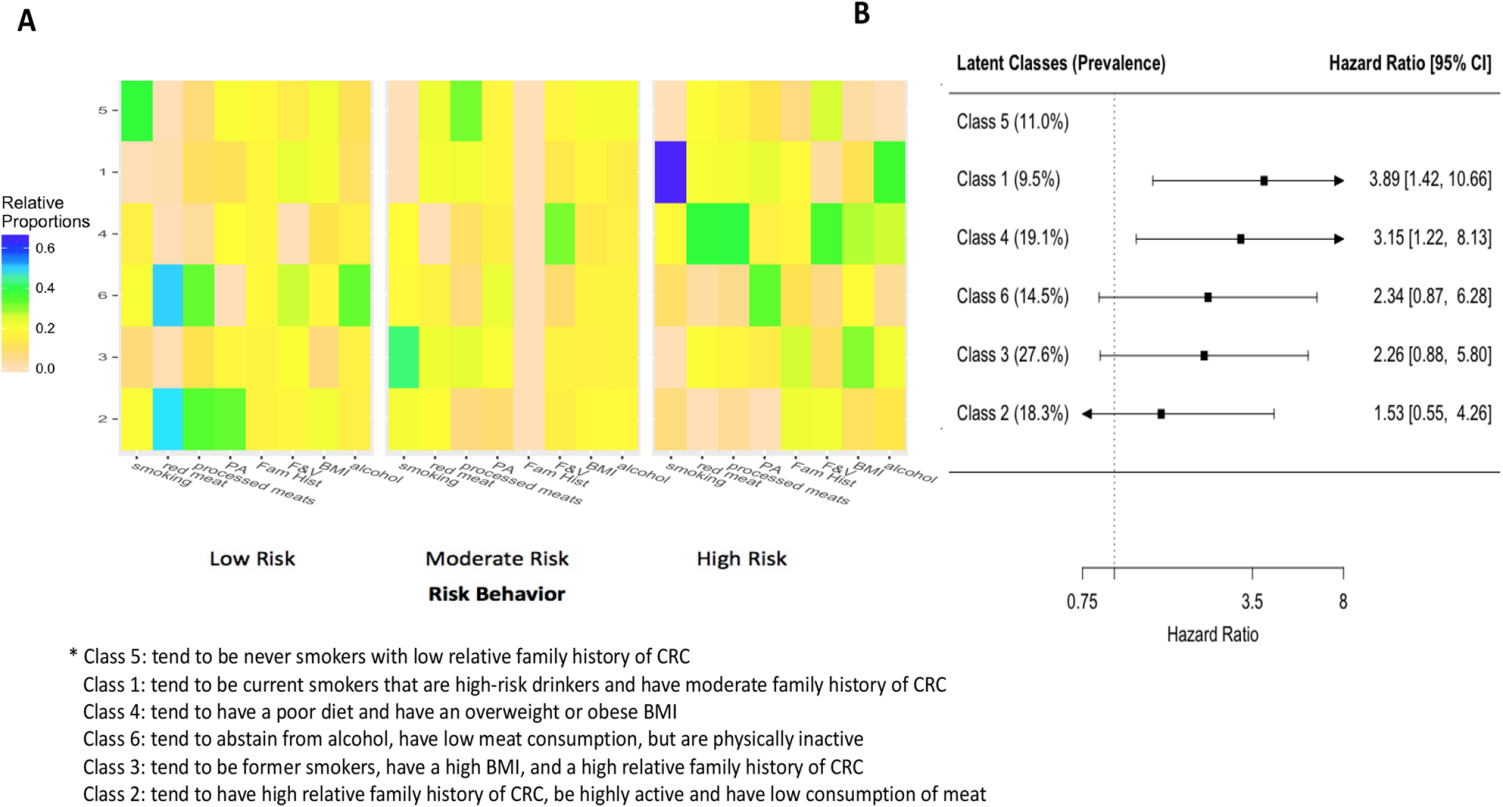
Latent class analysis of lifestyle-related colorectal cancer risk behaviours among men and the association of these classes with the risk of developing colorectal cancer. Panel A depicts the relative proportion of participants that belong in each category for each risk behaviour and family history of colorectal cancer in each class. For each risk behaviour, the left is the low risk category and the right is the high risk category. Rows represent the latent classes and each column represents a category for a risk behaviour. Abbreviations: BMI = Body Mass Index, F & V = fruits and vegetables, Fam Hist = Family history of colorectal cancer, PA = physical activity. Panel B depicts the risk (hazard ratios) of colorectal cancer for each class compared to the lowest risk class (class 5) adjusted for age, ethnicity, household income, and education.

**Figure 3:**
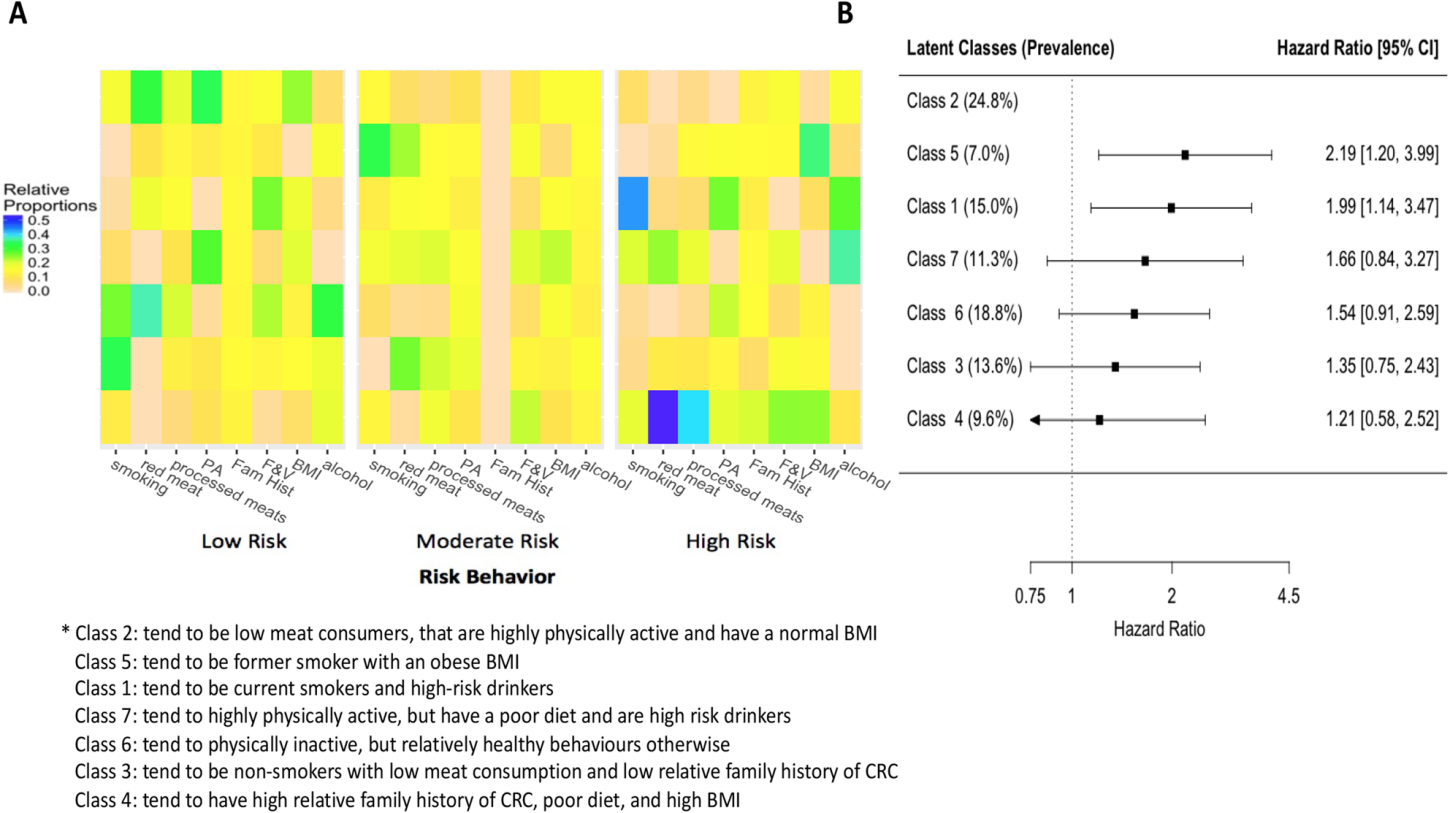
Latent class analysis of lifestyle-related colorectal cancer risk behaviours among women and the association of these classes with the risk of developing colorectal cancer. Panel A depicts the relative proportion of participants that belong in each category for each risk behaviour and family history of colorectal cancer in each class. For each risk behaviour, the left is the low risk category and the right is the high risk category. Rows represent the latent classes and each column represents a category for a risk behaviour. Abbreviations: BMI = Body Mass Index, F & V = fruits and vegetables, Fam Hist = Family history of colorectal cancer, PA = physical activity. Panel B depicts the risk (hazard ratios) of colorectal cancer for each class compared to the lowest risk class (class 2) adjusted for age, ethnicity, household income, and education.

The behavioural profiles of each sex-specific latent class and the risk of developing CRC compared to the low risk class are displayed in Fig. 2 and 3, respectively. The absolute probabilities for each risk behaviour for each latent class by sex are presented in Supplemental Tables 4 and 5. For men, the low risk group (class 5) contained 11.0% of the male study sample and tended to consist of never smokers with low relative family history of CRC. Compared to the low risk group, the risk of CRC was 3.89 (95% CI, 1.42–10.67) for the class that tended to consist of current smokers that were high-risk drinkers and have moderate family history of CRC (class 1) and 3.15 (95% CI, 1.22–8.12) for the group that tended to have a poor diet and an overweight or obese BMI (class 4). Compared to the low risk group, the risk was 2.34 (95% CI, 0.96–10.05) for the group that tended to have a low consumption of meat, abstain from alcohol and be physically inactive (class 6); 2.26 (95% CI, 0.88–5.80) for the group that tended to be former smokers, have a high BMI and have a high relative family history of CRC (class 3); and 1.53 (95% CI, 0.55–4.26) for the group that tended to have a high relative family history of CRC, be highly active and have low consumption of red and processed meat (class 2).

For women, the low risk group (class 2) contained 24.8% of the female study sample and tended to consist of low meat consumers that are highly physically active and have a normal BMI. Compared to the low risk group, the risk of CRC was 2.19 (95% CI, 1.20–3.98) for the class that tended to consist of former smokers with an obese BMI (class 5) and 1.99 (95% CI, 1.14–3.47) for the group that tended to be current smokers and high-risk drinkers BMI (class 1). Compared to the low risk group, the risk of CRC was 1.66 (95% CI, 0.80–3.11) for the group that tended to be highly physically active, but have a poor diet and be high-risk drinkers (class 7); 1.54 (95% CI, 0.92–2.61) for the group that tended to be physically inactive, but had relatively healthy behaviours otherwise (class 6); and 1.35 (95% CI, 0.75–2.43) for the group that tended to have a low relative family history of CRC, be non-smokers and have low consumption of red and processed meat (class 3). Interestingly, the group that had the highest relative family history of CRC, poor diet, and high BMI only had an increased risk of 1.21 (95% CI, 0.58–2.51) compared to the low risk group.

There were several noteworthy differences between the risk behaviour groups and corresponding CRC risk between men and women. First, the proportion of women in the low risk group was more than double that of the low risk group for men. For women, the low risk group tended to consist of low meat consumers that are highly physically active and have a normal BMI, while for men this group tended to consist of never smokers with a low relative family history of CRC. Despite a larger low risk group, the risk of CRC tended to be higher in behavioural profiles for men than for women, and a family history of CRC tended to play a stronger role in class formation for men than for women. The groups with the highest risk for men tended to consist of current smokers that were high-risk drinkers and tended to consist of individuals with a poor diet and a high BMI. In contrast, for women the groups with the highest risk for tended to consist of former smokers with an obese BMI and individuals that are current smokers and high-risk drinkers.

### Sensitivity analyses

When restricting analyses to participants that were followed for greater than a year or greater than two years, the effect estimates were similar in magnitude to the main analysis for the overall and female latent classes, but were slightly stronger effect estimates for the men-specific latent classes (Supplemental Table 6). The results for the sensitivity analyses using more precise measures for smoking and obesity are presented in Supplemental Figs. 1, 2, and 3. For the overall analysis and analysis for men, the classes and the associated risks of developing CRC were similar to the main analysis. For women, a new high-risk group emerged that consisted of women that tended to be heavy smokers, eat red meat, engage in low levels of physical activity, and have an obese BMI and waist circumference out of the normal range. Compared to the low risk class, the risk of developing CRC for this latent class was 3.05 (95% CI, 1.64–5.68).

## Discussion

In this long-term prospective study of Albertans, we conducted an exploratory latent class analysis to elucidate common risk behavioural profiles of lifestyle-related CRC risk behaviours and assessed how these profiles were related to the risk of developing CRC. Of the seven identified profiles, the lowest risk group did not exhibit exclusively healthy behaviours – despite tending to be never smokers and having a normal BMI, they also tended to eat insufficient fruit and vegetables. In addition, the analysis did not identify a single highest risk CRC group, but four moderate risk groups with distinct engagement in multiple negative behaviours. With the exception of obesity, which is a strong risk factor for CRC, engaging in multiple poor lifestyle-behaviours without offsetting it with healthy behaviours conferred the greatest risk of CRC. In sex-stratified analyses, common behaviour profiles included current smokers that are also high-risk drinkers and former smokers that have an obese BMI.

A surprising finding of this study, was that there was not a *bona fide* high-risk group, but four groups with risk of CRC two to three times greater than the low risk group. Instead of a group that engaged in a multitude of these behaviours, groups tended to engage in a few poor behaviours and not engage in others – moderating their overall risk of CRC. The highest risk group consisted of individuals that were current smokers, high-risk drinkers and inactive, which are all moderate risk factors for CRC. The next class tended to consist of individuals with a poor diet (low fruit and vegetable and high red and processed meat consumption). While diet-related exposures are weaker risk factors for CRC, this result may indicate interactions of these risk factors. One of the other high-risk groups tended to consist of individuals with an overweight BMI that were inactive, but had a healthy diet. Given that this group tended to have a healthy diet, this could indicate some interaction between BMI and physical inactivity, which has been demonstrated before^21^. Only one of the high risk groups had one poor behaviour, which was a group that tended to consist of individuals with an obese BMI, highlighting the importance of BMI in the etiology of CRC^5^. Despite the importance of obesity, the risks associated for the groups with multiple negative behaviours were higher than the single risk factors, which highlights the potential importance of interventions targeting multiple behaviours simultaneously. Interestingly, the two groups that did not have significantly greater risk of developing CRC compared to the low risk group tended to engage in high levels of physical activity, which could be evidence of the importance of physical activity for offsetting some other unhealthy behaviours. Indeed, a recent meta-analysis showed that physical activity was more protective against colorectal cancer if individuals have an overweight or obese BMI^21^.

In sex stratified analyses, two common profiles emerged – current smokers that were also high-risk drinkers, and former smokers that had an obese BMI. In addition to being common across sexes, these two profiles tend to be common among other studies^9,11^ and should be targeted with multifactorial interventions. Outside of these profiles, behavioural patterns tend to be sex-specific, which have important implications for prevention programs. The risk associated with developing CRC for behavioural groups tended to be higher among men than for women. This can be explained partly by the groups among men having more unhealthy behaviours, but is also likely a product of genetic factors that predispose men to have a higher risk of CRC in the general population. In addition, among men, the group with the highest relative family history of CRC had the greatest amount of healthy behaviours, which was not the case among women. There is some evidence that individuals with family history of cancer tend to mitigate other risk factors^22^. Men are at a higher risk of CRC than women in general and therefore a family history of CRC may be more incentive for men than women to engage in healthy behaviours. Therefore, family history could be an important factor to take into account in terms of designing population-based prevention programs.

This type of approach can provide valuable information for potential interventions that could have the greatest impact at the population level – assuming that the interventions are effective at behavioural modification. For instance, the highest risk group consists of current smokers that are high-risk drinkers and are inactive. Given that smoking cessation can lead to weight gain in the short-term, smoking cessation interventions that promote physical activity would likely have the biggest impact on disease risk at the population level. That is, the intervention would positively modify two risk behaviours without any negative consequences. An intervention focused solely on smoking would positively modify one behaviour, but could also indirectly promote a negative risk factor – a higher BMI.

In this study, all behaviours were assumed to meaningfully contribute to risk based on previous meta-analyses that have examined the independent effects of these single risk factors^5–8^. This analysis tried to move one step further to understanding behavioural patterns and their impacts on disease risk more holistically. As we observed, few CRC risk factors exist in isolation, so these more complete considerations of multiple factors are warranted. Since only two of the established risk factors for CRC were statistically significant in this study, our analysis was limited by a small sample of a single cohort. Given that behaviours are not homogeneous within classes, this misclassification could lead to potentially misleading results in a study with a low number of cases – depending on the distribution of cases among mis-specified participants. This misclassification could have led to the observation of four high-risk groups with similar risk of CRC. However, the majority of groups had risks that were slightly higher than their expected multiplicative risks, which provides some evidence that class misspecification did not have a major influence on the results. Overall, this approach requires replication in an independent population with a larger sample size.

The primary strength of this study is that we identified unique behavioural profiles and assessed the risk of these profiles with the development of colorectal cancer, which could have utility for population level cancer prevention. In addition, this approach allows for a clean assessment of the risk of disease associated with multiple risk behaviours that is difficult to determine with standard interaction analyses. We performed the latent class analysis using three categories for each risk factor, which is not typically done and allowed for the elucidation of more specific classes. Analytically, we used attained age as the time-scale in our proportional hazards models, since enrolment in a study is not a meaningful start time and using length of follow-up can lead to residual confounding by age. In addition, we censored any individual that developed another cancer, since treatment for the cancer could increase the risk of subsequent CRC. Other strengths of this study include the prospective study design, long follow-up, and little missing data.

All information on personal risk behaviours was self-reported, and therefore social desirability bias and measurement error may have influenced our results. While the aim of the questionnaires is to represent average exposure over adult years, all the risk behaviour groups were based on baseline data at a single time point. Therefore, they may not represent lifetime exposure that could influence disease risk and could be subject to change between baseline and last follow-up. Future studies with full exposure histories or time-varying exposure should utilize this approach to establish comprehensive risk behaviour groups or more precise estimates of disease risk. The cut-offs used to categorize each risk behaviour was based on established guidelines, which may have led to the misclassification of more extreme behavioural patterns. However, classification in this way allows for future comparisons with other populations and are likely the most impactful for interpretation of a large population. The clusters are not completely homogeneous and do show some variation, which influences the interpretability of cancer risk. However, the general group profiles tend to be quite distinct from one another, such that they can provide an indication of the average risk of one groups’ profile compared to another. We did not have data on personal history of colorectal polyps, which is a risk factor for CRC and this may have biased some of the effect estimates in this study. Finally, our sample only included 26,000 individuals, which limits how distinct groups can be and how precise estimates of the risk of CRC could be. The behavioural profiles identified in this study may only be generalizable to Alberta and potentially only a subset of Albertans, since individuals that participate in these types of cohort studies tend to be systematically different from individuals that do not. Specifically, this cohort tends to be older, have a higher socioeconomic status, higher BMI, less smoking, and consume more alcohol than the general Alberta population^23,24^.

There are several large ongoing cohort studies with data on modifiable lifestyle behaviours throughout the world. For Canada specifically, Alberta's Tomorrow Project is only one of five ongoing cohort studies, collectively recruiting over 300,000 Canadians^25^. Data from these cohorts and others could be utilized to identify common behavioural profiles with more precision and nuance. That is, with larger sample sizes, more specific groups can be identified and their risk of disease better quantified. In addition, larger sample sizes would allow for stratified analyses by key factors, such as age groups, income groups, family history of cancer, and urban or rural residence. This approach could be widely applied to other cancer sites and other chronic disease, such as cardiovascular disease. Disease-specific lifestyle behaviours or a wider set of behaviours, including some unestablished risk factors, could be utilized to inform disease-specific or more general multifactorial intervention programs.

Given that behaviours tend to cluster in unique and distinct ways, targeting combinations of risk behaviours simultaneously and specific to the combination could lead to greater prevention of CRC on a population level. Furthermore, the messages targeted at a single risk factor may not be adequate to meaningfully reduce CRC risk at the population level. Future studies with larger sample sizes and examining the risk of behavioural profiles on multiple diseases are required to inform on multifactorial intervention programs.

## Methods

### Data source

In this study, we utilized baseline data from ATP, a prospective cohort study of adults aged 35 to 69 years with no previous history of cancer, residing in the Canadian province of Alberta^24^. Beginning in the year 2000, a total of 31,121 individuals enrolled in ATP and completed the Health and Lifestyle Questionnaire (HLQ), which captured demographic information, family history of cancer, personal health history, and several lifestyle behaviours. In addition to the HLQ, 26,538 of the original participants completed the Canadian Diet History Questionnaire-I (DHQ) and the Past Year Total Physical Activity Questionnaire (PYTPAQ)^26^, which captured detailed dietary information, and accelerometer-validated physical activity information for different domains (employment/volunteer based, transportation-related, household-related, and recreational based activities), respectively.

### Exposures of interest

A set of established^5–8^ modifiable lifestyle risk factors for CRC were identified from ATP: overweight or obese BMI, inadequate levels of physical activity, excess consumption of red and processed meat, inadequate fruit and vegetable consumption, alcohol consumption, and tobacco smoking. These specific health behaviours were utilized since all of these behaviours are broadly applicable to the development of cancer and other chronic diseases. Standard or common measurements for health behaviours were utilized so that the methods could be replicated and utilized at the population level. To facilitate interpretation, each of the identified risk factors were categorized into three distinct levels based on established cut points or Canadian and international guidelines. If the distribution of an exposure did not align with an established cut point or guideline, we chose categories that had approximately equal proportion of participants in each category. Tobacco smoking was categorized as never, former, and current smoker. BMI was categorized using the World Health Organization (WHO) classification of normal weight (< 25 kg/m^2^), overweight (25–30 kg/m^2^), and obese (≥ 30 kg/m^2^). Alcohol consumption was categorized based on the World Cancer Research Fund (WCRF)/American Institute for Cancer Research (AICR) recommendations for cancer prevention, which advise ≤ 1 drink/day for women and ≤ 2 drinks/day for men. ATP participants were classified into abstainers, low risk drinkers (under the guidelines), and high risk drinkers (consumption greater than the guidelines). Recreational physical activity was categorized based on Canadian and international guidelines, which recommend 150–300 min of moderate to vigorous physical activity per week. Participants were classified into low activity (under the guideline), moderate activity (met guidelines), and high activity (exceeded guidelines). The recommended consumption of fruits and vegetables is greater than four serving of each per day. Participants were classified into low consumption (did not meet the recommendation for either fruits or vegetables), moderate consumption (met the recommendation for either fruits or vegetables), and high consumption (met the recommendation for both fruits and vegetables). The recommended guideline for consumption of red and processed meat is less than one serving (75 g or 36 oz) per week for each. Since a considerable portion of the ATP participants exceeded these guidelines, we categorized participants based on servings of meat per day. For red meat we classified participants into low (less than 3 servings per week), moderate (3–6 servings per week), and high (greater than 6 servings per week). For processed meat we classified participants into low (less than one serving per week), moderate (1–2 servings per week), and high (greater than 2 servings per week).

### Latent class analysis

Clustering of CRC risk factors among participants in ATP was investigated using exploratory LCA. LCA is a clustering analysis that allows for the identification of homogeneous, mutually exclusive groups or classes of individuals within a heterogeneous population^13^. Each latent class can be characterised by its estimated prevalence in the total study sample and the probability of individuals within that class exhibiting each risk factor. To account for uncertainty in class membership the model assigns each individual a posterior probability of class membership. To conduct the LCA, we used the PROC LCA package in SAS^5^. Since male sex and family history of CRC are associated with increased risk for CRC and both have the potential to impact the adoption of risk behaviours, we conducted latent class analyses stratified by sex and including family history of CRC in the class formation to determine if there were any patterns of behaviour that were sex-specific and related to family history of CRC.

To determine the smallest and most interpretable number of clusters, we began with a two-class model and successively increased the number of classes by one, fitting a new LCA model to the data at each step until we identified the simplest model that provided adequate fit determined with fit statistics. In selecting a final model solution, we examined the balance between both the BIC and AIC, as the BIC tends to underestimate the number of classes present and the AIC tends to overestimate. In addition to fit statistics, our model selection considered the prevalence of participants in each class, since adequately sized classes were important for downstream analyses. Moreover, the model selected also considered the elucidation of a distinctly low colorectal cancer risk class, since our estimates of cancer risk depend on comparisons of unique risk behaviour profiles with healthy patterns of behaviour. After selecting the most appropriate number of classes, Bayes theorem was used to compute each participant’s posterior probability of membership in each latent class. Participants were assigned to the class that had the maximum posterior probability. To assess the general performance of the maximum posterior probability, we calculated the mean and median maximum posterior probabilities for each class, as well as the probabilities for being in other classes. To simplify the interpretation of the characteristics of each class, we standardized each class to a sample of 1000 and calculated relative probabilities across classes for each category of each risk behaviour. A high relative probability can be interpreted as a high probability of the class consisting of individuals that engage in that behaviour compared to the average of the study sample. We then created a heat map of these relative probabilities for each class to identify the unique behavioural profiles of each class.

### Association of latent class groups with the risk of colorectal cancer

Incident cases of primary CRC were identified through data linkage with the Alberta Cancer Registry (ACR) up to December 2017. Participant follow-up time was estimated from their exact age at entry to their exact age at cancer diagnosis (first-site cancer diagnosed), or at the end of follow-up (their exact age at the time of data linkage with ACR in December 2017). If a participant developed a cancer other than CRC they were censored at the exact age at diagnosis. Cox proportional hazards models, with attained age as the time-scale, were used to estimate the HR between the “lowest risk latent class” and each of the other latent classes after adjusting for sex (male/female), ethnicity (white/other), family history of CRC (yes/no), highest level of education (high school or less/some post-high school education/post-high school certificate or degree), and total household income ($0 to $49,999 or $50,000 to $99,999 or ≥ $100,000). The proportional hazards assumption was tested using the cumulative sums of martingale-based residuals method, which tests that each variable in the final model meets the proportional hazard assumption^27^. Analyses were also conducted for the latent classes that were derived from sex-specific LCA modelling. For all analyses, a two-tailed P-value of < 0.05 was considered statistically significant. We did not correct for multiple comparisons due to low study power and a limited number of comparisons being performed. All statistical analyses were performed using SAS 9.4.

### Sensitivity analyses

We conducted sensitivity analyses where participants that were followed for less than one year or two years were excluded to minimize the potential of reverse causation (i.e., participant’s behaviours being influenced by existing, yet undiagnosed cancers). As a sensitivity analyses, we conducted new latent class analyses with more precise measures for smoking and obesity. For smoking we categorized participants into three categories: non-smoker, smoker with less than 15 pack-years smoked, and smoker with greater than or equal to 15 pack-years smoked. For obesity we used a combination of BMI and waist circumference. Waist circumference was categorized into two categories: normal (< 102 cm for men and < 88 cm for women) and high (≥ 102 cm for men and ≥ 88 cm for women) and then were combined with the standard BMI categories. We then categorized participants into three categories: normal BMI, overweight BMI but normal waist circumference, overweight BMI and high waist circumference or obese BMI.

### Ethics statement

All participants signed an informed consent form to be included in the study and also consented to the linkage of their questionnaire responses to administrative databases, including the Alberta Cancer Registry (ACR). Ethics approval for recruitment and data collection was obtained from the former Alberta Cancer Board Research Ethics Committee and the University of Calgary Conjoint Health Research Ethics Board. All methods were performed in accordance with relevant guidelines and regulations. The guidelines of strengthening the reporting of observational studies in epidemiology (STROBE) were followed for reporting of the results of this study^28^.

## Supplementary information

Supplementary Information.

## Data Availability

All associated code, protocols, additional results and/or aggregated data from this study are available from the corresponding author upon reasonable request. Additional access to individual-level data is available in accordance with the Health Information Act of Alberta and the data access guidelines of Alberta’s Tomorrow Project at https://myatp.ca/. The data from this study included all participants from Phase I of cohort data collection accessed under accession code Brenner-2016–04.
