# Supplementary material for: Combinations of modifiable lifestyle behaviours in relation to colorectal cancer risk in Alberta’s Tomorrow Project
Source: Sci Rep. 2020 Nov 25;10:20561. doi: 10.1038/s41598-020-76294-w (PMC7689485; doi:10.1038/s41598-020-76294-w)
Supplement: Supplementary file 1 — Supplementary Information. [file 41598_2020_76294_MOESM1_ESM.docx]

**Combinations of modifiable lifestyle behaviours in relation to colorectal cancer risk in Alberta’s Tomorrow Project**

Dylan E. O’Sullivan^1^, Amy Metcalfe^2,3,4^, Troy W.R. Hillier^1^, Will D. King^1^, Sangmin Lee^2^, Joy Pader^5^, Darren R. Brenner^2,5,6^

Affiliations:

1. Department of Public Health Sciences, Queen’s University, Kingston, ON

2. Department of Community Health Sciences, University of Calgary, AB

3. Department of Obstetrics & Gynecology, University of Calgary, AB

4. Department of Medicine, University of Calgary, AB

5. Department of Cancer Epidemiology and Prevention Research, Alberta Health Services, Calgary, AB

6. Department of Oncology, University of Calgary, Calgary, AB

**Supplemental Tables and Figures**

**Supplemental Table 1.** Characteristics of male participants in the Alberta Tomorrow Project and the risk of developing CRC for each variable.

|  | **Cases (n=119)** | **Controls (n=9,773)** | **Hazard Ratio*** |
| --- | --- | --- | --- |
|  | **n (%)** | **n (%)** | **(95% CI)** |
| **Follow-up time (years)** |  |  |  |
| Mean (SD) | 8.09 (4.11) | 12.83 (3.71) |  |
| **Age at baseline (years)** |  |  |  |
| Mean (SD) | 57.2 (8.15) | 50.98 (9.14) |  |
| **BMI** |  |  |  |
| Normal | 13 (10.92) | 2257 (23.09) | ref |
| Overweight | 53 (44.54) | 4823 (49.35) | **1.71 (0.93-3.15)** |
| Obese | 53 (44.54) | 2693 (27.56) | **2.85 (1.54-5.29)** |
| **Fruit and vegetable consumption** |  |  |  |
| Did not meet guideline for fruit nor vegetable | 16 (13.45) | 1365 (13.97) | ref |
| Met guideline for fruit or vegetable | 41 (34.45) | 3946 (40.38) | 0.84 (0.47-1.51) |
| Met guideline for both fruit and vegetable | 62 (52.10) | 4462 (45.66) | 1.26 (0.72-2.22) |
| **Alcohol Consumption** |  |  |  |
| Abstainer | 11 (9.24) | 1302 (13.32) | ref |
| Low risk (under guidelines) | 85 (71.43) | 7154 (73.20) | 1.56 (0.83-2.94) |
| High risk (greater than guidelines) | 23 (19.33) | 1317 (13.48) | **2.19 (1.05-4.59)** |
| **Processed Meat** |  |  |  |
| Less than 1 serving per week | 42 (35.29) | 3814 (39.03) | ref |
| 1-2 servings per week | 28 (23.53) | 2518 (25.76) | 1.06 (0.65-1.74) |
| Greater than 2 servings per week | 49 (41.18) | 3441 (35.21) | 1.41 (0.90-2.21) |
| **Red Meat** |  |  |  |
| Less than 3 servings per week | 24 (31.93) | 1819 (18.61) | ref |
| 3-6 servings per week | 34 (28.57) | 3413 (34.92) | 0.74 (0.43-1.27) |
| Greater than 6 servings per week | 61 (51.26) | 4541 (46.46) | 1.00 (0.65-1.74) |
| **Recreational Physical Activity** |  |  |  |
| Less than 150 min per week | 38 (31.93) | 3122 (31.95) | ref |
| 150-300 min per week | 28 (23.53) | 2101 (21.50) | 1.21 (0.74-1.98) |
| Greater than 300 min per week | 53 (44.54) | 4550 (46.56) | 1.19 (077-1.82) |
| **Tobacco Smoking** |  |  |  |
| Never | 29 (24.37) | 4101 (41.99) | ref |
| Former | 63 (52.94) | 3912 (40.03) | 1.40 (0.89-2.19) |
| Current | 27 (22.69) | 1757 (17.98) | **1.77 (1.03-3.04)** |
| **Ethnicity** |  |  |  |
| Caucasian | 114 (95.80) | 8828 (90.33) | ref |
| Other | 5 (4.20) | 945 (9.67) | 0.56 (0.23-1.37) |
| **Household Income** |  |  |  |
| $0-$49,999 | 42 (35.29) | 2322 (23.76) | ref |
| $50,000-$99,999 | 46 (38.66) | 4324 (44.24) | 0.78 (0.51-1.20) |
| >=$100,000 | 31 (26.05) | 3127 (32.00) | 0.88 (0.54-1.43) |
| **Highest level of education** |  |  |  |
| High school or less | 42 (35.29) | 2429 (24.85) | ref |
| Some post-high school | 21 (17.65) | 1790 (18.32) |  |
| Degree | 56 (47.06) | 5554 (56.83) |  |
| **Family History of CRC** |  |  |  |
| No | 100 (84.03) | 9062 (92.72) | ref |
| Yes | 19 (15.97) | 711 (7.28) | **1.94 (1.18-3.17)** |

*Hazard ratios are mutually adjusted

**Supplemental Table 2.** Characteristics of female participants in the Alberta Tomorrow Project and the risk of developing CRC for each variable.

|  | **Cases (n=148)** | **Controls (n=16,420)** | **Hazard Ratio*** |
| --- | --- | --- | --- |
|  | **n (%)** | **n (%)** | **(95% CI)** |
| **Follow-up time (years)** |  |  |  |
| Mean (SD) | 7.54 (4.33) | 12.71 (3.59) |  |
| **Age at baseline (years)** |  |  |  |
| Mean (SD) | 56.71 (8.92) | 50.78 (9.21) |  |
| **BMI** |  |  |  |
| Normal | 41 (27.70) | 6678 (40.67) | ref |
| Overweight | 61 (41.22) | 5441 (33.14) | 1.47 (0.99-2.19) |
| Obese | 46 (31.08) | 4301 (26.19) | 1.28 (0.83-1.98) |
| **Fruit and vegetable consumption** |  |  |  |
| Did not meet guideline for fruit nor vegetable | 23 (15.54) | 2572 (15.66) | ref |
| Met guideline for fruit or vegetable | 48 (32.43) | 6028 (36.71) | 0.86 (0.52-1.42) |
| Met guideline for both fruit and vegetable | 77 (52.03) | 7820 (47.62) | 1.07 (0.66-1.72) |
| **Alcohol Consumption** |  |  |  |
| Abstainer | 34 (22.97) | 2811 (17.12) | ref |
| Low risk (under guidelines) | 96 (64.86) | 11626 (70.80) | 0.83 (0.56-1.24) |
| High risk (greater than guidelines) | 18 (12.16) | 1983 (12.08) | 0.99 (0.55-1.79) |
| **Processed Meat** |  |  |  |
| Less than 1 serving per week | 98 (66.22) | 11102 (67.61) | ref |
| 1-2 servings per week | 33 (22.30) | 3101 (18.89) | 1.34 (0.89-2.01) |
| Greater than 2 servings per week | 17 (11.49) | 2217 (13.50) | 0.96 (0.56-1.65) |
| **Red Meat** |  |  |  |
| Less than 3 servings per week | 68 (45.95) | 7236 (44.07) | ref |
| 3-6 servings per week | 58 (39.19) | 6404 (39.00) | 1.03 (0.72-1.49) |
| Greater than 6 servings per week | 22 (14.86) | 2780 (16.93) | 0.91 (0.54-1.53) |
| **Recreational Physical Activity** |  |  |  |
| Less than 150 min per week | 65 (43.92) | 5955 (36.27) | ref |
| 150-300 min per week | 45 (30.41) | 4005 (24.39) | 1.15 (0.78-1.69) |
| Greater than 300 min per week | 38 (25.68) | 6460 (39.34) | 0.70 (0.46-1.06) |
| **Tobacco Smoking** |  |  |  |
| Never | 57 (38.51) | 7643 (46.55) | ref |
| Former | 58 (39.19) | 5973 (36.38) | 1.22 (0.84-1.76) |
| Current | 33 (22.30) | 2804 (17.08) | **1.55 (1.00-2.41)** |
| **Ethnicity** |  |  |  |
| Caucasian | 137 (92.57) | 14949 (91.04) | ref |
| Other | 11 (7.43) | 1471 (8.96) | 1.00 (0.54-1.86) |
| **Household Income** |  |  |  |
| $0-$49,999 | 81 (54.73) | 5737 (34.94) | ref |
| $50,000-$99,999 | 49 (33.11) | 6386 (38.89) | 0.74 (0.52-1.08) |
| >=$100,000 | 18 (12.16) | 4267 (26.17) | 0.50 (0.30-0.86) |
| **Highest level of education** |  |  |  |
| High school or less | 54 (36.49) | 4894 (29.81) | ref |
| Some post-high school | 32 (21.62) | 3594 (21.89) | 0.99 (0.64-1.53) |
| Degree | 62 (41.89) | 7932 (48.31) | 1.08 (0.74-1.58) |
| **Family History of CRC** |  |  |  |
| No | 127 (85.81) | 14993 (91.31) | ref |
| Yes | 21 (14.19) | 1427 (8.69) | 1.42 (0.89-2.26) |

*Hazard ratios are mutually adjusted

**Supplemental Table 3.** The absolute probabilities for each risk behaviour for each latent class from the overall latent class model.

|  | **Class 1** | **Class 2** | **Class 3** | **Class 4** | **Class 5** | **Class 6** | **Class 7** |
| --- | --- | --- | --- | --- | --- | --- | --- |
|  | 13.9% | 10.2% | 19.1% | 20.5% | 16.4% | 11.8% | 8.1% |
| **BMI** |  |  |  |  |  |  |  |
| Normal | 0.1840 | 0.6390 | 0.5259 | 0.3439 | 0.0060 | 0.3528 | 0.4360 |
| Overweight | 0.4731 | 0.3610 | 0.3594 | 0.3824 | 0.2306 | 0.6127 | 0.4011 |
| Obese | 0.3428 | 0.0000 | 0.1147 | 0.2736 | 0.7634 | 0.0345 | 0.1629 |
| **Fruit and vegetable consumption** |  |  |  |  |  |  |  |
| Did not meet guideline for fruit nor vegetable | 0.2495 | 0.2684 | 0.1711 | 0.0417 | 0.1543 | 0.1686 | 0.0205 |
| Met guideline for fruit or vegetable | 0.6572 | 0.3958 | 0.2992 | 0.2087 | 0.4249 | 0.4521 | 0.3164 |
| Met guideline for both fruit and vegetable | 0.0934 | 0.3358 | 0.5297 | 0.7496 | 0.4208 | 0.3793 | 0.6631 |
| **Alcohol Consumption** |  |  |  |  |  |  |  |
| Abstainer | 0.1069 | 0.1251 | 0.1356 | 0.2792 | 0.2529 | 0.0038 | 0.0558 |
| Low risk (under guidelines) | 0.6862 | 0.8749 | 0.7477 | 0.6836 | 0.7379 | 0.6632 | 0.6142 |
| High risk (greater than guidelines) | 0.2068 | 0.0000 | 0.1167 | 0.0372 | 0.0092 | 0.3330 | 0.3299 |
| **Processed Meat** |  |  |  |  |  |  |  |
| Less than 1 serving per week | 0.0345 | 0.4417 | 0.8600 | 0.8859 | 0.4235 | 0.5722 | 0.4495 |
| 1-2 servings per week | 0.2565 | 0.3536 | 0.0846 | 0.0786 | 0.3498 | 0.2570 | 0.2852 |
| Greater than 2 servings per week | 0.7090 | 0.2047 | 0.0554 | 0.0356 | 0.2267 | 0.1708 | 0.2652 |
| **Red Meat** |  |  |  |  |  |  |  |
| Less than 3 servings per week | 0.0000 | 0.0193 | 0.8849 | 0.7612 | 0.0384 | 0.0268 | 0.1149 |
| 3-6 servings per week | 0.0000 | 0.6786 | 0.1151 | 0.2388 | 0.6061 | 0.7567 | 0.5617 |
| Greater than 6 servings per week | 1.0000 | 0.3021 | 0.0000 | 0.0000 | 0.3555 | 0.2165 | 0.3234 |
| **Recreational Physical Activity** |  |  |  |  |  |  |  |
| Less than 150 min per week | 0.3094 | 0.1918 | 0.0000 | 0.7042 | 0.5106 | 0.0010 | 0.7013 |
| 150-300 min per week | 0.2055 | 0.3280 | 0.1427 | 0.2958 | 0.2134 | 0.2452 | 0.2420 |
| Greater than 300 min per week | 0.4851 | 0.4802 | 0.8573 | 0.0000 | 0.2761 | 0.7538 | 0.0568 |
| **Tobacco Smoking** |  |  |  |  |  |  |  |
| Never | 0.3550 | 0.9915 | 0.5054 | 0.5375 | 0.4525 | 0.1271 | 0.0107 |
| Former | 0.3760 | 0.0000 | 0.3948 | 0.3304 | 0.5177 | 0.7114 | 0.1708 |
| Current | 0.2690 | 0.0085 | 0.0997 | 0.1320 | 0.0299 | 0.1616 | 0.8185 |

**Supplemental Table 4.** The absolute probabilities for each risk behaviour for each latent class from the male latent class model.

|  | **Class 1** | **Class 2** | **Class 3** | **Class 4** | **Class 5** | **Class 6** |
| --- | --- | --- | --- | --- | --- | --- |
|  | 9.5% | 18.3% | 27.6% | 19.1% | 11.0% | 14.5% |
| **BMI** |  |  |  |  |  |  |
| Normal | 0.3228 | 0.3284 | 0.0963 | 0.1926 | 0.3369 | 0.2592 |
| Overweight | 0.5130 | 0.5247 | 0.4713 | 0.4108 | 0.6340 | 0.4864 |
| Obese | 0.1642 | 0.1469 | 0.4324 | 0.3966 | 0.0290 | 0.2544 |
| **Fruit and vegetable consumption** |  |  |  |  |  |  |
| Did not meet guideline for fruit nor vegetable | 0.0170 | 0.1660 | 0.0782 | 0.2773 | 0.1975 | 0.0432 |
| Met guideline for fruit or vegetable | 0.3012 | 0.3349 | 0.3554 | 0.7212 | 0.3751 | 0.1854 |
| Met guideline for both fruit and vegetable | 0.6818 | 0.4991 | 0.5664 | 0.0014 | 0.4274 | 0.7714 |
| **Alcohol Consumption** |  |  |  |  |  |  |
| Abstainer | 0.0747 | 0.1190 | 0.1213 | 0.1188 | 0.0880 | 0.2620 |
| Low risk (under guidelines) | 0.6387 | 0.7787 | 0.7097 | 0.6739 | 0.9120 | 0.7094 |
| High risk (greater than guidelines) | 0.2865 | 0.1023 | 0.1690 | 0.2073 | 0.0000 | 0.0286 |
| **Processed Meat** |  |  |  |  |  |  |
| Less than 1 serving per week | 0.2378 | 0.8358 | 0.2865 | 0.0686 | 0.1701 | 0.8056 |
| 1-2 servings per week | 0.3488 | 0.0982 | 0.3843 | 0.1514 | 0.5104 | 0.1031 |
| Greater than 2 servings per week | 0.4134 | 0.0660 | 0.3292 | 0.7799 | 0.3195 | 0.0913 |
| **Red Meat** |  |  |  |  |  |  |
| Less than 3 servings per week | 0.0113 | 0.5800 | 0.0035 | 0.0000 | 0.0000 | 0.5958 |
| 3-6 servings per week | 0.4553 | 0.4182 | 0.4883 | 0.0000 | 0.4880 | 0.3436 |
| Greater than 6 servings per week | 0.5334 | 0.0018 | 0.5083 | 1.0000 | 0.5120 | 0.0606 |
| **Recreational Physical Activity** |  |  |  |  |  |  |
| Less than 150 min per week | 0.4858 | 0.0000 | 0.2969 | 0.3114 | 0.2556 | 0.6997 |
| 150-300 min per week | 0.2152 | 0.1029 | 0.2599 | 0.1921 | 0.2133 | 0.3003 |
| Greater than 300 min per week | 0.2990 | 0.8971 | 0.4432 | 0.4965 | 0.5311 | 0.0000 |
| **Tobacco Smoking** |  |  |  |  |  |  |
| Never | 0.0000 | 0.5152 | 0.1783 | 0.3990 | 1.0000 | 0.5338 |
| Former | 0.0000 | 0.3891 | 0.8217 | 0.3412 | 0.0000 | 0.3310 |
| Current | 1.0000 | 0.0958 | 0.0000 | 0.2598 | 0.0000 | 0.1352 |
| **Family history of CRC** |  |  |  |  |  |  |
| Yes | 0.9287 | 0.9084 | 0.8949 | 0.9319 | 0.9759 | 0.9526 |
| No | 0.0713 | 0.0916 | 0.1051 | 0.0681 | 0.0241 | 0.0474 |

**Supplemental Table 5.** The absolute probabilities for each risk behaviour for each latent class from the female latent class model.

|  | **Class 1** | **Class 2** | **Class 3** | **Class 4** | **Class 5** | **Class 6** | **Class 7** |
| --- | --- | --- | --- | --- | --- | --- | --- |
|  | 15.0% | 24.8% | 13.6% | 9.6% | 7.0% | 18.8% | 11.3% |
| **BMI** |  |  |  |  |  |  |  |
| Normal | 0.4528 | 0.6032 | 0.3701 | 0.1875 | 0.0000 | 0.3179 | 0.5003 |
| Overweight | 0.3503 | 0.3468 | 0.2862 | 0.2213 | 0.1301 | 0.3682 | 0.4976 |
| Obese | 0.1968 | 0.0501 | 0.3437 | 0.5912 | 0.8699 | 0.3139 | 0.0022 |
| **Fruit and vegetable consumption** |  |  |  |  |  |  |  |
| Did not meet guideline for fruit nor vegetable | 0.0000 | 0.1883 | 0.1894 | 0.2874 | 0.1713 | 0.0782 | 0.2270 |
| Met guideline for fruit or vegetable | 0.2031 | 0.3233 | 0.3898 | 0.6343 | 0.4835 | 0.2132 | 0.5917 |
| Met guideline for both fruit and vegetable | 0.7969 | 0.4884 | 0.4208 | 0.0783 | 0.3451 | 0.7086 | 0.1813 |
| **Alcohol Consumption** |  |  |  |  |  |  |  |
| Abstainer | 0.0544 | 0.0855 | 0.2257 | 0.2421 | 0.1993 | 0.3978 | 0.0000 |
| Low risk (under guidelines) | 0.6906 | 0.7682 | 0.7743 | 0.6624 | 0.7455 | 0.6022 | 0.6633 |
| High risk (greater than guidelines) | 0.2551 | 0.1463 | 0.0000 | 0.0955 | 0.0552 | 0.0000 | 0.3367 |
| **Processed Meat** |  |  |  |  |  |  |  |
| Less than 1 serving per week | 0.6805 | 0.8663 | 0.5327 | 0.2249 | 0.5890 | 0.9074 | 0.4454 |
| 1-2 servings per week | 0.2372 | 0.0825 | 0.3301 | 0.2701 | 0.2298 | 0.0547 | 0.3185 |
| Greater than 2 servings per week | 0.0823 | 0.0512 | 0.1372 | 0.5050 | 0.1812 | 0.0379 | 0.2361 |
| **Red Meat** |  |  |  |  |  |  |  |
| Less than 3 servings per week | 0.4370 | 0.7680 | 0.0045 | 0.0029 | 0.2405 | 0.9050 | 0.0000 |
| 3-6 servings per week | 0.4803 | 0.2320 | 0.7985 | 0.0611 | 0.7199 | 0.0950 | 0.5880 |
| Greater than 6 servings per week | 0.0828 | 0.0000 | 0.1970 | 0.9361 | 0.0395 | 0.0000 | 0.4120 |
| **Recreational Physical Activity** |  |  |  |  |  |  |  |
| Less than 150 min per week | 0.7777 | 0.0011 | 0.4034 | 0.5912 | 0.4572 | 0.6445 | 0.0290 |
| 150-300 min per week | 0.2180 | 0.1651 | 0.3365 | 0.1861 | 0.2562 | 0.2978 | 0.2819 |
| Greater than 300 min per week | 0.0043 | 0.8338 | 0.2601 | 0.2227 | 0.2867 | 0.0577 | 0.6891 |
| **Tobacco Smoking** |  |  |  |  |  |  |  |
| Never | 0.0515 | 0.4683 | 0.9493 | 0.3333 | 0.0000 | 0.7304 | 0.2033 |
| Former | 0.3484 | 0.4289 | 0.0000 | 0.4023 | 1.0000 | 0.2303 | 0.5159 |
| Current | 0.6001 | 0.1028 | 0.0507 | 0.2644 | 0.0000 | 0.0393 | 0.2808 |
| **Family history of CRC** |  |  |  |  |  |  |  |
| Yes | 0.9211 | 0.9145 | 0.9527 | 0.8635 | 0.9003 | 0.9006 | 0.9059 |
| No | 0.0789 | 0.0855 | 0.0473 | 0.1365 | 0.0997 | 0.0994 | 0.0941 |

**Supplemental Table 6.** Sensitivity analysis restricting to participants that were

followed for greater than one year and two years

|  | **No latency** | **One year latency** | **Two year latency** |
| --- | --- | --- | --- |
|  | **HR (95% CI)** | **HR (95% CI)** | **HR (95% CI)** |
| **Overall** |  |  |  |
| Class 7 | 2.87 (1.43-5.77) | 3.07 (1.49-6.33) | 2.85 (1.37-5.91) |
| Class 1 | 2.48 (1.27-4.83) | 2.46 (1.22-4.94) | 2.34 (1.16-4.72) |
| Class 5 | 2.46 (1.28-4.70) | 2.51 (1.27-4.94) | 2.42 (1.23-4.77) |
| Class 4 | 2.34 (1.23-4.45) | 2.44 (1.25-4.77) | 2.30 (1.17-4.53) |
| Class 6 | 1.73 (0.85-3.49) | 1.73 (0.83-3.63) | 1.58 (0.75-3.34) |
| Class 3 | 1.56 (0.79-3.06) | 1.66 (0.82-3.36) | 1.56 (0.76-3.16) |
|  |  |  |  |
| **Men** |  |  |  |
| Class 1 | 3.89 (1.42-10.66) | 4.55 (1.50-13.77) | 4.55 (1.50-13.77) |
| Class 4 | 3.15 (1.22-8.13) | 3.67 (1.29-10.47) | 3.67 (1.29-10.47) |
| Class 6 | 2.34 (0.87-6.28) | 2.97 (1.01-8.74) | 2.84 (0.96-8.40) |
| Class 3 | 2.26 (0.88-5.80) | 2.69 (0.95-7.62) | 2.70 (0.95-7.65) |
| Class 2 | 1.53 (0.55-4.26) | 1.80 (0.59-5.54) | 1.67 (0.54-5.20) |
|  |  |  |  |
| **Women** |  |  |  |
| Class 5 | 2.19 (1.20-3.99) | 1.97 (1.06-3.65) | 1.90 (0.99-3.65) |
| Class 1 | 1.99 (1.14-3.47) | 2.00 (1.14-3.49) | 1.82 (1.00-3.30) |
| Class 7 | 1.66 (0.84-3.27) | 1.65 (0.88-3.10) | 1.45 (0.73-2.86) |
| Class 6 | 1.54 (0.91-2.59) | 1.42 (0.83-2.42) | 1.45 (0.83-2.53) |
| Class 3 | 1.35 (0.75-2.43) | 1.29 (0.71-2.33) | 1.40 (0.76-2.57) |
| Class 4 | 1.21 (0.58-2.52) | 1.21 (0.58-2.53) | 1.32 (0.63-2.77) |

**Supplemental Figure 1.** Latent class analysis of lifestyle-related colorectal cancer risk behaviours and the association of these classes with the risk of developing colorectal cancer. Panel A depicts the relative proportion of participants that belong in each category for each risk behaviour in each class. For each risk behaviour, the left is the low risk category and the right is the high risk category. Rows represent the latent classes and each column represents a category for a risk behaviour. Abbreviations: BMI = Body Mass Index, F & V = fruits and vegetables, PA = physical activity, WC = waist circumference. Panel B depicts the risk (hazard ratios) of colorectal cancer for each class compared to the lowest risk class (class 2) adjusted for age, sex, ethnicity, household income, education, and family history of CRC.

**Supplemental Figure 2.** Latent class analysis of lifestyle-related colorectal cancer risk behaviours among men and the association of these classes with the risk of developing colorectal cancer. Panel A depicts the relative proportion of participants that belong in each category for each risk behaviour and family history of colorectal cancer in each class. For each risk behaviour, the left is the low risk category and the right is the high risk category. Rows represent the latent classes and each column represents a category for a risk behaviour. Abbreviations: BMI = Body Mass Index, F & V = fruits and vegetables, Fam Hist = Family history of colorectal cancer, PA = physical activity, WC = waist circumference. Panel B depicts the risk (hazard ratios) of colorectal cancer for each class compared to the lowest risk class (class 2) adjusted for age, ethnicity, household income, and education.

**Supplemental Figure 3.** Latent class analysis of lifestyle-related colorectal cancer risk behaviours among men and the association of these classes with the risk of developing colorectal cancer. Panel A depicts the relative proportion of participants that belong in each category for each risk behaviour and family history of colorectal cancer in each class. For each risk behaviour, the left is the low risk category and the right is the high risk category. Rows represent the latent classes and each column represents a category for a risk behaviour. Abbreviations: BMI = Body Mass Index, F & V = fruits and vegetables, Fam Hist = Family history of colorectal cancer, PA = physical activity, WC = waist circumference. Panel B depicts the risk (hazard ratios) of colorectal cancer for each class compared to the lowest risk class (class 2) adjusted for age, ethnicity, household income, and education.
